# Coupling with COVID: The Role of Dyadic Coping in Relationship Satisfaction and Psychological Distress during the COVID-19 Pandemic

**DOI:** 10.1177/0192513X211030028

**Published:** 2022-08

**Authors:** Michelle T. Leonard, Charles Giraud, Christen Abraham

**Affiliations:** 1 Department of Behavioral Sciences, 14711 University of Michigan – Dearborn, Dearborn, MI, USA

**Keywords:** intimate relationships, family health, dyadic relationship/quality/satisfaction, dyadic coping, gender and family

## Abstract

Models of dyadic coping suggest that facing a stressful situation, such as the COVID-19 pandemic, with one’s partner to meet their needs is associated with positive outcomes. This study explored dyadic coping and its association with relationship satisfaction and distress in the time of the COVID-19 pandemic. Data were collected online from 564 participants. Participants completed measures of dyadic coping, relationship satisfaction, COVID anxiety, and OCD, and asked to describe their experience in an open-ended question. Results showed that experiences were quite polarized. Significant gender differences and differences for couples with/without children were noted for distress and relationship satisfaction. There was a significant interaction between dyadic coping and relationship satisfaction for women when predicting COVID OCD; however, post-hoc analysis showed that this interaction was only significant for women with children. The potential exponential burden that female couple members may face during COVID-19 as well as implications for intervention, are discussed.

## Introduction

The COVID-19 crisis is clearly a unique stressor that has impacted the health of millions around the globe. There have been, understandably, considerable efforts to understand the physiological impacts of the disease, but comparatively lesser research has been done on the psychological and social consequences of this pandemic (Xiang et al., 2020). At this juncture, it is critical to understand how individuals and couples alike are coping with these stressful circumstances. Studies have shown that coping with stress is not only an important predictor of individual outcomes, but continued stress has been shown to be associated with poor couple outcomes such as couples’ communication problems, low relationship satisfaction, and eventual higher likelihood for divorce (Scott, Rhoades, Stanley, Allen, & Markman, 2013). Therefore, the current study is aimed at examining how dyadic coping is associated with distress in married or cohabiting relationships associated with the COVID-19 pandemic and relationship satisfaction.

A focus on couples relationships is logical given that in the United States, over half of individuals are in committed romantic relationships and roughly 50% of the American population are married (Scott, Schelar, Manlove, & Cui, 2009; Wildsmith, Manlove, Steward-Streng, & Cook, 2013). Moreover, a number of direct benefits of relationships have been identified in the literature including general happiness and financial security (Waite and Gallagher, 2001) for both heterosexual and LGBTQ couples (Wienke and Hill, 2009). Benefits of marriage also link to the health realm and research has found that individuals in couples have better immune functioning and lesser disability (e.g., Kiecolt-Glaser and Newton, 2001; Koball, Moiduddin, Henderson, Goesling, & Besculides, 2010). Although dyadic relationships in general show benefits to health, these seem to be dependent on the quality of the relationship. Numerous studies have shown that couples with higher levels of relationship satisfaction have the best outcomes for well-being (Kiecolt-Glaser and Newton, 2001; Røsand, Slinning, Eberhard-Gran, Røysamb, & Tambs, 2012; Schmaling, Afari, Barnhart, & Buchwald,1997).

During the current pandemic, couples are now dealing with new financial struggles, educational responsibilities, and balancing work requirements while at home, in addition to individual-level stressors. A working paper by Carlson, Petts, and Pepin (2020) highlights the increases in household responsibilities for families and exacerbation of already existing gender divides among some households. Moreover, couples have to take their own individual health, as well as the health of their family members, into consideration for many activities that could pose a risk (e.g., shopping for essentials, gathering for support, and returning to work).

Couched in Lazarus and Folkman’s (1984) transactional model of stress, Bondenmann’s model of dyadic coping highlights stress management through a couples lens. This type of coping focuses on the shared experience of stress and coping by both partners of a couple. It can broadly be defined as “the interplay between the stress signals of one partner and the coping reactions of the other, a genuine act of shared coping” (Revenson, Kayser, & Bodenmann, 2005, p. 4). Bodenmann (2008) outlines several important factors involved in the dyadic coping process. Common dyadic coping involves coping strategies when both partners are experiencing a stressful event, as is the case with COVID-19. Supportive coping refers to coping efforts directed to one couple member when that partner is primarily concerned with the stressor (e.g., one couple member a frontline worker). Lastly, delegated coping refers to efforts by one partner to take over tasks/duties to alleviate stress on the other (e.g., alternating child care responsibility during work at home times).

Dyadic coping has also been shown to be directly related to relationship satisfaction. For instance, in a sample of heterosexual dating couples, Papp and Witt (2010) found that positive and negative aspects of dyadic coping influenced relationship satisfaction above and beyond that of individual coping strategies. In a study that tracked couples over a period of days, dyadic coping was shown to impact immune functioning to stressors where those partners in low dyadic coping relationships had increased immune reactivity (Reed, O'Connor, Pace, Raison, & Butler, 2017). In addition to the link between dyadic coping and relationship satisfaction, studies have found positive effects of dyadic coping for both physical and mental health outcomes.

In terms of mental health outcomes, early reports on mental health and the COVID-19 pandemic outcomes show considerable rates of psychological distress, namely, symptoms of anxiety. Research utilizing an online sample of respondents, most of whom had never sought treatment for anxiety, Lee (2020) found that his measure of coronavirus anxiety was associated with hopelessness, passive suicidal ideation, and increased coping with substances. Safety behaviors and contamination fears of infection have increased since the pandemic began (Knowles and Olatunji, 2020). A study of health care workers in China showed increases in symptoms of depression, insomnia, somatization, and OCD (Zhang et al., 2020). Similarly, Rajkumar (2020) found that depressive and anxiety symptoms were found among approximately 25% of those who did and did not work on the front lines.

Based on the potential role that dyadic coping can have on the health and well-being of couples in times of stress, it is imperative that we have an understanding of these processes during this unprecedented time. Therefore, the goal of the current project was to examine dyadic coping responses among romantic dyads amidst the COVID-19 pandemic. We will also explore how dyadic coping is associated with COVID anxiety and distress. Examination of this topic now is critical as there have been some studies (Bondenmann et al., 2014) that suggest that self-directed approaches using low-cost technologies can show improvements in couples functioning, which in turn could ultimately lead to specific interventions during this unique time to support couples.

## Methods

### Participants

Participants for the current project were recruited through a Qualtrics online survey that was disseminated using snowball recruitment techniques. The Qualtrics online survey was anonymized in order to scrub IP addresses from participants who participated. The PI and research staff shared the link for the study through various contacts and social media outlets with instructions to continue to share with others who might be eligible. This type of data collection method has been used in countless studies in the past with positive results and can be especially helpful to target a wide range of potential participants (e.g., Browne, 2005). In order to be eligible, participants were required to be 18 years of age or older, currently in a cohabiting relationship, and must be able to complete the survey in English.

Data collection was open for a 2 week time period. During the 2 weeks, the survey collected at least partial data from 694 potential participants. Of these data sets, 110 were not complete (with at least 90% of data completed) leaving the sample size of 564. Of these remaining participants, there were a number who answered negatively to at least one of the eligibility requirements. Three participants stated that they were not 18 years old, 28 of the participants indicated that they were not living with their partner at the time of the study, and one said that they could not complete the survey in English. This leaves the total sample size at 530 individuals.

### Measures

#### Demographics information

In order to understand the demographic characteristics of the sample, participants were asked several demographic questions. These questions focused on age, gender identity, relationship status, relationship start date, ethnicity/race, sexual orientation, children, political affiliation, occupation, work status, and income. Participants were also asked about how strictly they have followed stay at home/safety measures associated with the coronavirus.

#### Relationship satisfaction

Couples Satisfaction Index (Funk & Rogge, 2007): The couples satisfaction index was developed using item response theory to provide a more fine tailored measurement of relationship satisfaction. Items on the measure were derived from commonly used measures of relationship satisfaction that had previously been used in the literature. The measure has different versions; however, for the current study, the four-item version was chosen for brevity. In its validation sample, the measure showed strong reliability with an alpha of .84 (Funk and Roggee, 2007). It should be noted that two participants in the study skipped each item on this measure so analysis with relationship satisfaction includes a sample size of 528. Alpha for this measure for the current study was excellent (a = .93).

#### Dyadic coping

The Dyadic Coping Inventory (DCI: Bodenmann, 2008; Ledermann et al., 2010) is a 37-item instrument designed to measure perceived communication and dyadic coping (supportive, delegated, negative, and joint) that occurs in close relationships when one or both partners are stressed. Questions are aimed at an individual’s own attempts to regulate stress in their partner and also common strategies that a couple may use to help one another cope. Items on the DCI are rated on a Likert scale ranging from 1 (“very rarely”) to 5 (“very often”). Initial validation studies of the DCI show high internal consistency (Bodenmann, 2008). The DCI has established cut-off scores to group responses into below average, normal, and above average ranges. If an item on this measure was missed by a participant, a mean item score was replaced. There was not any item on this measure that had more than two participants miss the item. Reliability for the current study was good (a =.82).

#### COVID-19-related psychological distress

Coronavirus Anxiety Scale (Lee, 2020a, 2020b): The Coronavirus Anxiety Scale is a short five-item measure that is designed to measure common symptoms of anxiety that can be associated with exposure to COVID-19-related information or thoughts. In its initial validation study, the scale showed strong internal consistency. In the current study, the reliability for this measure was good (a = .82).

The Obsession with COVID-19 Scale (Lee, 2020a, 2020b) was developed to assess dysfunctional thought patterns associated with COVID-19. This four-item scale uses a Likert-type format with anchors at 0 (not at all) and 4 (nearly every day over the last 2 weeks). A score of 7 or more has been suggested as a cutoff for significant dysfunctional thoughts associated with COVID-19. Reliability for the measure in the current study was adequate (a = .77).

#### Qualitative questions for couples

The final question of the online survey asked participants to share their thoughts/experiences about couples coping with COVID in an open-ended fashion. There were 69 of the participants that chose to write a response to the question. In the responses, there were 12 men, 54 women, and three LBGTQ participants. Study team members then coded the responses into several categories: working from home (e.g., the participant is currently working from home during the pandemic), partner working from home (e.g., the participant’s partner is currently working from home during the pandemic), child care (e.g., the participant is taking care of their child/children they may have at home), sick/loved one care (e.g., the participant is taking care of a family member that has health problems and is in need of assistance), general partner misunderstanding (e.g., the participant seems to have a form of miscommunication or is not on the same page with their values and/or their viewpoint on a current situation with their partner), participant COVID positive (e.g., the participant has been tested positive for COVID-19), partner COVID positive (e.g., the participant’s partner has been tested positive for COVID-19), general COVID practical stress (e.g., the participant is experiencing a great amount of anxiousness regarding the COVID-19 outbreak), participant health (e.g., the participant is worried about or has an existing health condition), partner health concerns (e.g., the participant is worried about their partners existing health condition), happy to be home (e.g., the participant has an overall content fullness in being home during the pandemic), familial/external stressors (e.g., the participant is experiencing external stressors from outside the home), bored (e.g., the participant is experiencing a spiritless and dull time during the current pandemic), and financial stress (e.g., the participant is experiencing financial hardship and distress).

### Procedure

Prior to any data collection, the study was reviewed for participant safety through the university’s IRB. As noted above, the participants were recruited through social media and online platforms, starting with the PI and research study staff. The link to the research study was provided in posts/messages about the study for potential participants to link directly to the study. When potential participants clicked the study link, they were directed to a Qualtrics online survey. The first page of the survey included the study’s consent form that outlines the purpose of the study, the potential risks and benefits, and general study procedures. The participants were asked to check a box to indicate their consent to the study prior to any data collection being done. Participants were not able to enter further into the survey without this response. After participants provided consent, they were directed to three questions to ensure that they were eligible to participate (i.e., over age 18, cohabiting with a partner, and able to complete the survey in English). If any of these questions were answered in a way that would indicate that the participant is ineligible, they were directed to the debriefing/“thank you” message. If the participant was eligible to participate, they would proceed to the study measures, which all were delivered through the Qualtrics online survey. It should be noted that participants were not required to answer any specific question in order to proceed with the study. This technique was chosen so that participants would not be forced to answer a question that they are not comfortable answering.

## Results

On average, participants completed the survey in 25.42 minutes (SD = 162.36 minutes; range 2.52–3041.35 minutes). Demographic data for the participants can be found in Table 1. As can be seen, the majority of the participants identified as white females. The sample was predominantly heterosexual and married. Fifty-three percent of the sample had children living at home with an average of 2.03 (SD = .998) children. Participants in the study were also well-educated and the majority were employed full-time at the time of the study.

**Table 1.** Demographic Characteristics of Participants.

	Males % (*N*)	Females % (*N*)	Total % (*N*)
Race			
Asian/Pacific Islander	4 (5.3%)	8 (1.9%)	
Black/African American	1 (1.3%)	13 (3.1%)	
Latinx	2 (2.6%)	9 (2.1%)	
Middle Eastern and North African	3 (3.9%)	21 (5.0%)	
Native American or Indian	0 (0%)	4 (1.0%)	
White/Caucasian	68 (89.5%)	380 (90.5%)	513 (100%)
Sexual orientation			
Heterosexual	67 (88.2%)	379 (90.2%)	
Gay/lesbian	2 (2.6%)	7 (1.7%)	
Bisexual	2 (2.6%)	27 (6.4%)	
Pansexual	3 (3.9%)	3 (0.7%)	
None of the above	1 (1.3%)	1 (0.2%)	
Prefer not to answer	1 (1.3%)	3 (0.7%)	496 (96.7%)
Relationship status			
Engaged	5 (6.6%)	22 (5.2%)	
Married	55 (72.4%)	323 (76.9%)	
In a committed relationship	16 (21.1%)	75 (17.9%)	496 (96.7%)
Highest level of education			
High school diploma/GED	3 (3.9%)	13 (3.1%)	
Some college	19 (25.0%)	57 (13.6%)	
Associate’s degree	4 (5.3%)	31 (7.4%)	
Bachelor’s degree	27 (35.5%)	125 (29.8%)	
Master’s degree	14 (18.4%)	150 (35.7%)	
PhD	9 (11.8%)	41 (9.8%)	493 (96.1%)
Employment status			
Full time (<40 hours per week)	55 (72.4%)	284 (67.6%)	
Unemployed at this time	14 (18.4%)	81 (19.3%)	
Part time (>40 hours per week)	7 (9.2%)	55 (13.1%)	496 (96.7%)
Household income			
Below $10,000	1 (1.3%)	2 (0.5%)	
$10,000–$24,999	2 (2.6%)	4 (1.0%)	
$25,000–$49,999	9 (11.8%)	36 (8.6%)	
$50,000–$74,999	19 (25.0%)	55 (13.1%)	
$75,000–$99,999	7 (9.2%)	76 (18.1%)	
Over $100,000	34 (44.7%)	221 (52.6%)	
Prefer not to answer	4 (5.3%)	26 (6.2%)	513 (100%)

*Note.* The majority of participants identified with a binary gender; therefore, data were presented for males and females only.

There were no gender differences in psychological distress (COVID anxiety or OCD) or relationship variables (dyadic coping or relationship satisfaction) based on demographic variables of employment status or income. There were significant differences in study variables across genders. Specifically women reported more COVID anxiety (t = −2.61 (494), *p* <.001; M = 7.01, SD = 2.49) than men (M = 6.22, SD = 1.95) and COVID OCD (t = −2.52 (494), *p* <.01; M = 6.76, SD = 2.58) than men (M = 5.95 SD = 2.58). There were also significant differences amongst all study variables except COVID anxiety for participants who reported that there were children (under the age of 18) in the home or not. There were not significant differences in relationship satisfaction or dyadic coping based on gender. Several significant differences among those participants who reported children and those who did not were identified. As can be seen in Table 2, participants without children reported increased COVID OCD but not anxiety. In addition, participants with children reported significant lower levels of relationship satisfaction and dyadic coping.

**Table 2.** Differences among Study Variables for Participants with/without Children.

		M (SD)	t(df)	SE	*p*
CO_OCD	Children	6.241 (2.327)	−3.593 (505)	.230	.00
	No children	7.067 (2.781)			
CO_ANX	Children	6.773 (2.345)	−1.234 (505)	.217	.22
	No children	7.042 (2.559)			
Rel Sat	Children	15.093 (4.097)	−3.709 (503)	.354	.00
	No children	16.405 (3.814)			
Dyad Cope	Children	129.920 (16.741)	−3.465 (505)	1.509	.00
	No children	135.150 (17.195)			

*Note.* Rel Dur = Relationship Duration, Rel Sat =Relationship Satisfaction as measured by the Couples Satisfaction Inventory, Dyad Cope = Dyadic coping as measured by the Dyadic Coping Scale, CO-OCD = COVID-19 OCD as measured by the Obsession with COVID-19 Scale, CO_ANX = COVID-19 Anxiety as measured by the Coronavirus Anxiety Scale.

After examining the demographic data, the first step in data analysis was to examine responses to the open-ended question. There were a total of sixty-nine (approximately 14% of participants in total) participants who answered this question. The most common categories reported by participants included: happy to be home (52%), general COVID stress (22%), working from home (20%), partner working from home (19%), and child care (12%). The remaining frequency of categories can be found in Table 3. The data were then examined in terms of the association between these qualitative responses and relationship functioning and psychological distress. Based on these responses it was determined that the number of categories that were endorsed would be combined in a total stressor score and the “happy at home” response group would be examined independently.

**Table 3.** Qualitative Data Summary.

Category	% Endorsing (n)
**Work from home_Personal**	**20.3% (14)**
**Work from home_Partner**	**18.8% (8)**
**Caregiving_Children**	**18.8% (8)**
Caregiving_Ill Other	5.8% (4)
Partner Misunderstandings	7.2% (2)
COVID Partner	2.9% (2)
**COVID Stress/Anxiety**	**21.7% (15)**
Health Concerns Personal	7.2% (5)
Health Concerns Partner	4.3% (3)
**Happy to be Home**	**52.2% (36)**
External Stressor for Family	5.8% (4)
Bored at Home	1.4% (1)
Financial Stress	2.9% (2)

*Note. N* = 69. Top five most frequent responses are bolded.

There was a significant difference in the scores on relationship satisfaction as measured by the CSI for those who reported being happy at home (M = 17.19, SD = 2.83) and those that did not report this in their qualitative response (M = 14.65, SD = 4.29; t (66) = 2.906, *p* <.01). Similarly, there was a difference between those who reported being “happy at home” on dyadic coping (M = 138.43, SD = 16.08) and those that did not report this in their qualitative response (M = 129.33, SD = 20.03; t (67) = 2.089, *p* <.05). Results showed that there was a significant negative correlation between the summed stressor score and relationship satisfaction (r = −.315, *p* <.01) and dyadic coping (r = −.299, *p* < .05); however, the association between the qualitative response summed score was not significantly associated with either COVID anxiety (r = .204, *p* = .09) or COVID OCD symptoms (r = .042, *p* = .73).

To examine dyadic coping and its association with relationship satisfaction and psychological distress, correlations were conducted. Results of these correlations can be seen in Table 4. There was a significant negative association between age and COVID-related psychological distress (anxiety [r = −.14, *p* <.01] and OCD [r = −.12, *p* <.01]) and relationship variables (satisfaction [r = −.10, *p* <.01] and dyadic coping [r = −.09, *p* <.05]) and positively with relationship duration (r = .73, *p* < .001). Relationship duration was significantly negatively associated with COVID anxiety (r = −.09, *p* <.05) as well as dyadic coping 9r = −.16, *p* <.001). COVID anxiety was not associated with either relationship satisfaction or dyadic coping, but was significantly related to COVID OCD (r =.64, *p* <.001). COVID OCD was significantly negatively associated with relationship satisfaction (r = −.11, *p* <.05) and dyadic coping (r = −.09, *p* <.05) Relationship satisfaction was additionally positively associated with dyadic coping (r =.73, *p* <.001). An r to z transformation was conducted to compare these coefficients between the genders and this test showed a stronger relationship between these two variables for women (z = 2.68, *p* > .01). These correlations also show that relationship variables were not significantly associated with psychological distress for men. However, for women, relationship satisfaction and dyadic coping were significantly negatively associated with distress, namely, COVID OCD.

**Table 4.** Correlations among Study Variables.

	Age	Rel Dur	Rel Sat	Dyad Cope	COVID_OCD	COVID_Anx
Age	--	.707**	−.108*	−.064	−.132*	−.130**
Rel Dur	.792**	--	.098*	−.139*	−.081	−.093
Rel Sat	−.100	−.074	--	.767**	−.119*	−.007
Dyad Cope	−.224	−.287*	.587**	--	−.108*	.038
COVID_OCD	−.026	−.006	.030	.098	--	.634**
COVID_Anx	−.143	.057	−.040	.056	.654**	--

*Note.* Correlations above the diagonal are for women and below the diagonal are for men. Rel Dur = Relationship Duration, Rel Sat =Relationship Satisfaction as measured by the Couples Satisfaction Inventory, Dyad Cope = Dyadic coping as measured by the Dyadic Coping Scale, COVID_OCD = COVID-19 OCD as measured by the Obsession with COVID-19 Scale, COVID_Anx= COVID-19 Anxiety as measured by the Coronavirus Anxiety Scale.

** *p* <0.01.

* *p* <0.05.

**Figure 1.**
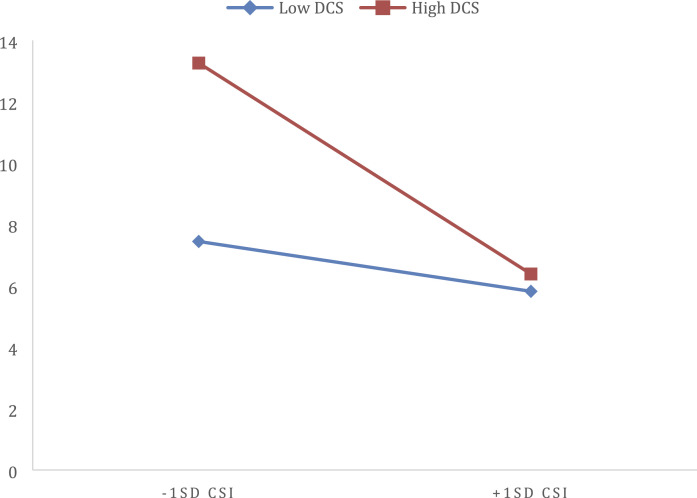
Interaction between Relationship Satisfaction and Dyadic Coping When Predicting COVID OCD Symptoms in Women.

We were then interested in examining how couples relationships impact distress, and based on the gender differences, including sample size, noted above, we choose to run analyses separate by gender. Two hierarchical regressions were conducted with the COVID anxiety and COVID OCD scales with simultaneous entry of relationship satisfaction and dyadic coping for men and women. Relationship satisfaction and dyadic coping or their interaction did not predict a significant proportion of variance in COVID anxiety scores for women (*R*^2^ = .004, *F* (1, 416) = .74, *p* > .05). For men, similar results were found such that there was not a significant main effect for either relationship variable or their interaction for COVID anxiety (*R*^2^ = .01, *F* (1, 74) = .41, *p* > .05). As can be seen in Table 5, there was significant interaction between relationship satisfaction and dyadic coping for women, but not for men, when predicting COVID OCD. Post hoc examination of this interaction using the method outlined by Holmbeck (2002) showed that at low levels of dyadic coping relationship satisfaction was not associated with COVID OCD (ß = −.064, t = −.841, *p* =.40); however, at high levels of dyadic coping, relationship satisfaction was negatively associated with COVID OCD (ß = −.284 t = −2.556, *p <.05*) (see Figure 1).

**Table 5.** Regressions Predicting COVID Anxiety and OCD Symptom for Men and Women.

Men								
	Variable	B	SE B	ß	t	F	R^2^	∆R^2^
Step 1								
	CSI	−.030	.104	−.042	−.291	.394	.011	
	DCS	.019	.023	.122	.850			
Step 2								
	CSI	−.124	.119	−.172	−.1043	1.077	.043	.032
	DCS	.035	.025	.217	1.401			
	CSI × DCS	−.009	.006	−.209	1.558			
Women								
	Variable	B	SE B	ß	t	F	R^2^	∆R^2^
Step 1								
	CSI	−.052	.048	−.083	−1.087	3.207*	.015	
	DCS	−.007	.011	−.048	−.634			
Step 2								
	CSI	−.110	.053	−.174	−2.071*	4.194**	.029	.14
	DCS	−.002	.011	−.016	−.203			
	CSI × DCS	−.004	.002	−.138	−2.468**			

*Note.* All variables were centered prior to regression analyses. CSI =Relationship Satisfaction as measured by the Couples Satisfaction Inventory, DCS = Dyadic coping as measured by the Dyadic Coping Scale.

** *p* <0.01.

* *p* <0.05.

## Discussion

The current study focused on couples’ experiences during the COVID-19 pandemic and how this influenced relationship functioning and psychological distress. Based on a stress response framework for couples coping (Bodenmann, 2008), dyadic coping was a particular focus for the current project, given the number of stressors and challenges for couples associated with the pandemic. Specifically, it was expected that dyadic coping would be associated with distress and relationship satisfaction; however, the study sought to explore how these variables interacted during an unprecedented stressful time for couples. Data were collected online from over 500 participants, mostly centered in the Midwestern region of the United States. Additionally, a qualitative analysis of couples experience was included to examine couples’ experiences during the COVID-19 pandemic.

Findings from the qualitative analysis suggest that overall couple members’ experiences were quite polarized. Approximately half of the participants indicated that they were “happy to be home” and this was subsequently associated with more positive relationship and psychological distress outcomes. The individuals who reported being “happy at home” may have occupational or financial resources to allow for this more positive transition during times of limited contact with others. Conversely, participants who did not include elements of being happy at home in their responses and indicated multiple stressors showed lower relationship satisfaction, dyadic coping and COVID anxiety. For these individuals, being home more often with their spouse/children may have served to exacerbate existing tension or it also may be that the pandemic has magnified issues related to job or financial security.

There were several significant gender differences noted between study variables and analysis by gender showed different effects of dyadic coping. Generally, women reported experiencing more COVID OCD and COVID anxiety than men. This finding may not be entirely surprising as a recent study of social media users (from which this convenience sample was gathered) showed that women were reporting more depression and anxiety during the time of the pandemic than men.

What was more interesting, however, was the association between dyadic coping and both relationship satisfaction and psychological distress. Dyadic coping was not directly associated with relationship satisfaction or psychological distress for men, but we saw both direct effects at the bivariate level and an interaction between dyadic coping and relationship satisfaction when predicting COVID OCD. For women, if they were not satisfied in their relationship, and would engage in higher levels of dyadic coping that their distress scores were quite high. However, when a couple member reported higher (+1SD) relationship satisfaction, dyadic coping efforts did not predict psychological distress. Other studies have found gender differences between the association of dyadic coping and relationship satisfaction, but not for individual well-being (Gmelch & Bodenmann, 2007).

**Figure 2.**
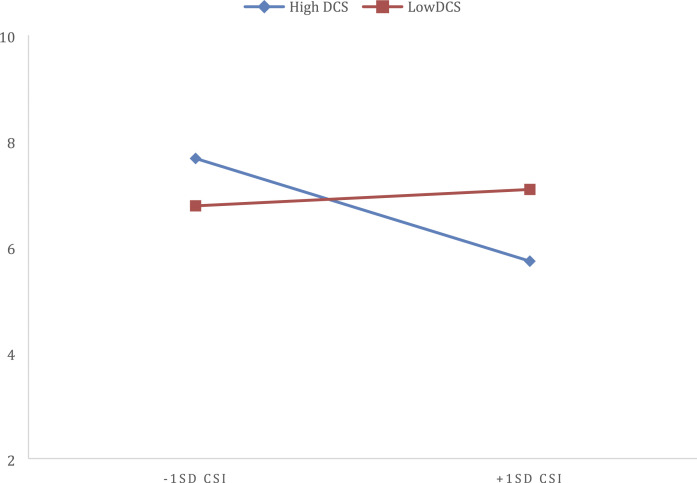
Interaction between Relationship Satisfaction and Dyadic Coping When Predicting COVID OCD Symptoms in Women with Children.

The findings from the current study were quite intriguing, especially this interaction for women. Based on some of the differences that were noted between participants who had children and those who did not, we decided to explore the significant interaction between dyadic coping and relationship satisfaction in women when predicting COVID OCD as a post hoc analysis. Using similar methodology to the original moderation analyses we ran separate analysis for women with and without children. Interestingly, this post hoc analysis showed that the interaction was significant only for those individuals with children (*R*^2^ = .04, *F* (2, 229) = 3.024, *p* > .05). Probing of this interaction showed that for women with children, engaging in low levels of dyadic coping relationship satisfaction was not associated with COVID OCD (ß = −.075, t = −.1.335, *p* =.18); however, at high levels of dyadic coping, relationship satisfaction was negatively associated with COVID OCD (ß = −.235 t = −2.800, *p <.001*) (see Figure 2).

The participants in the study were majority female, but these findings are nonetheless important to understand at a contextual level. Research conducted by Waddell et al. (2021) showed that during the COVID-19 pandemic, women took on more of the domestic work, such as parenting and housework, while men were more likely to be involved with paid work or personal matters during the span of the lockdown. Moreover, Fodor et al. (2021) found that while men and women both increased their contributions to child care at the same rate (35%) because women typically had already been contributing larger amounts of their time to child care, the contribution put forth by women during the pandemic grew significantly more than that of men, causing the gap between men and women’s working hours to grow. This study goes on to report that these findings were particularly apparent for women who were middle class, highly educated, and lived in a large city, with the greatest levels of gender inequality occurring amongst the highest educated couples. For heterosexual female couple members, having an additional workload of child care (which could include food preparation, household cleaning, disciplining, and assistance with virtual schooling) may be quite taxing on their coping resources. When they are then engaged in high levels of dyadic coping (to support themselves and their partner), this may tax their resources further. When a couple does not have a foundational bedrock of satisfaction to hold these additional burdens, it may lead to increased distress for women. This may also represent incongruence between couple members in terms of dyadic coping. Studies on dyadic coping with other stressors, such as chronic health issues, show that congruence is particularly important when examining the impact of dyadic coping. Meier and colleagues, recently found the effect of congruence was most important in predicting psychological distress in patients with breast cancer (Meier, Cairo Notari, Bodenmann, Revenson, & Favez, 2019).

Another important consideration for the current study is the concept of forced coexistence. In the event of COVID-19, many economic shutdowns forced couples to stay home for their safety and the safety of others. With such an excess amount of time being spent at home for an uncertain period of time couples members may feel more “stuck” with their partner. Recent research on forced coexistence and intimate partner violence (IPV) suggests that IPV has increased 23% since the pandemic lockdown under conditions of uncertainty and financial stress (Arenas-Arroyo, Fernández-Kranz, & Nollenberger, 2020). It can be concluded with the alignment of studies of forced coexistences, a polarizing experience is often reported.

The findings from this study highlight the need for targeting and tailoring interventions for couples, especially during this time of unprecedented matters. Especially, being able to focus on public health interventions that not only focus on maintaining a satisfying relationship, but which also relieves stress that female partners of heterosexual couples with children may experience due to forced coexistence. It may also be helpful for future research studies to examine levels of congruence or dyadic coping “matching” for couple members during the pandemic to see if interventions aimed at meeting the specific needs of each partner during this stressful unprecedented time.

Although the findings of the study are interesting, there are several notable limitations to the current study. Specifically, data were collected on the individual level and not the level of the dyad. It may be that changes within the dyad or couple-level perceptions of dyadic coping are more important predictors of relationship satisfaction or distress than that reported by an individual within a couple. Additionally, while cross-sectional studies allow us to understand associations among constructs, we cannot make any causal conclusions about relationship satisfaction and COVID-19. Also, this study was conducted approximately 3 months after the onset of the pandemic in the United States. The timing of the study may have impacted the results that were obtained and/or the generalizability of the findings to later time points in the pandemic. Another limitation to this study is the use of a convenience sample due to the employment of snowball sampling. Because of this technique, this study included participants who were predominantly white, lived in the Midwest region of the United States, well-educated, and had a higher household income than the average US household. Thus, this study’s population may not completely capture the diversity of the US population.

Despite these limitations, the study has several significant strengths that should be highlighted. Most notably, we have captured the couples’ experiences during the continued pandemic experience in the United States. Moreover, this is the only study of which we are aware that examines dyadic coping and couples satisfaction during the pandemic. The use of both qualitative and quantitative data in the current study is also important to note as this methodology allows for a “richer” picture of a couple members’ experience.

## References

[bibr1-0192513X211030028] Arenas-Arroyo E. Fernández-Kranz D. Nollenberger N. (2020). Can't leave you now! Intimate partner violence under forced coexistence and economic uncertainty. 10.1016/j.jpubeco.2020.104350 PMC9186438 35702337

[bibr2-0192513X211030028] Bodenmann G. (2008). Dyadisches coping inventar: Testmanual [dyadic coping inventory: Test manual]. Bern, Switzerland: Huber.

[bibr3-0192513X211030028] Bodenmann G. Hilpert P. Nussbeck F. W. Bradbury T. N. (2014). Enhancement of couples’ communication and dyadic coping by a self-directed approach: A randomized controlled trial. Journal of Consulting and Clinical Psychology, 82(4), 580. 24660673 10.1037/a0036356

[bibr4-0192513X211030028] Browne K. (2005). Snowball sampling: Using social networks to research non‐heterosexual women. International Journal of Social Research Methodology, 8(1), 47-60. DOI: 10.1080/1364557032000081663

[bibr5-0192513X211030028] Carlson D. L. Petts R. Pepin J. (2020). US couples’ divisions of housework and childcare during COVID-19 Pandemic.

[bibr6-0192513X211030028] Fodor É. Gregor A. Koltai J. Kováts E. (2021). The impact of COVID-19 on the gender division of childcare work in Hungary. European Societies, 23(sup1), S95-S110.

[bibr7-0192513X211030028] Funk J. L. Rogge R. D. (2007). Testing the ruler with item response theory: Increasing precision of measurement for relationship satisfaction with the Couples Satisfaction Index. Journal of Family Psychology, 21(4), 572. 18179329 10.1037/0893-3200.21.4.572

[bibr8-0192513X211030028] Gmelch S. Bodenmann G. (2007). Dyadisches coping in selbst-und fremdwahrnehmung als prädiktor für partnerschaftsqualität und befinden. Zeitschrift für Gesundheitspsychologie, 15(4), 177-186.

[bibr9-0192513X211030028] Holmbeck G. N. (2002). Post-hoc probing of significant moderational and mediational effects in studies of pediatric populations. Journal of Pediatric Psychology, 27(1), 87-96. 11726683 10.1093/jpepsy/27.1.87

[bibr10-0192513X211030028] Kiecolt-Glaser J. K. Newton T. L. (2001). Marriage and health: His and hers. Psychological Bulletin, 127(4), 472-503. DOI: 10.1037/0033-2909.127.4.472 11439708

[bibr11-0192513X211030028] Knowles K. A. Olatunji B. O. (2020). Anxiety and safety behavior usage during the COVID-19 pandemic: The prospective role of contamination fear. Journal of Anxiety Disorders, 77, 102323. DOI: 10.1016/j.janxdis.2020.102323 33137593 PMC7572316

[bibr12-0192513X211030028] Koball H. L. Moiduddin E. Henderson J. Goesling B. Besculides M. (2010). What do we know about the link between marriage and health? Journal of Family Issues, 31(8), 1019–1040. DOI: 10.1177/0192513X10365834

[bibr13-0192513X211030028] Lazarus R. S. Folkman S. (1984). Stress, appraisal, and coping. New York: Springer

[bibr14-0192513X211030028] Ledermann T. Bodenmann G. Gagliardi S. Charvoz L. Verardi S. Rossier J. Bertoni A. Iafrate R. (2010). Psychometrics of the dyadic coping inventory in three language groups. Swiss Journal of Psychology, 69, 201-212.

[bibr15-0192513X211030028] Lee S. A. (2020a). How much “Thinking” about COVID-19 is clinically dysfunctional? Brain, Behavior, and Immunity, 87, 97–98. DOI: 10.1016/j.bbi.2020.04.067. 32353520 PMC7185010

[bibr16-0192513X211030028] Lee S. A. (2020b). Coronavirus anxiety scale: A brief mental health screener for COVID-19 related anxiety. Death Studies, 44(7), 393–401. DOI: 10.1080/07481187.2020.1748481 32299304

[bibr17-0192513X211030028] Meier F. Cairo Notari S. Bodenmann G. Revenson T. A. Favez N. (2019). We are in this together—Aren’t we? Congruence of common dyadic coping and psychological distress of couples facing breast cancer. Psycho‐oncology, 28(12), 2374-2381. DOI: 10.1002/pon.5238 31600426

[bibr18-0192513X211030028] Papp L. M. Witt N. L. (2010). Romantic partners' individual coping strategies and dyadic coping: Implications for relationship functioning. Journal of Family Psychology, 24(5), 551–559. DOI: 10.1037/a0020836 20954765 PMC3220915

[bibr19-0192513X211030028] Rajkumar R. P. (2020). COVID-19 and mental health: A review of the existing literature. Asian Journal of Psychiatry, 52, 102066. DOI: 10.1016/j.ajp.2020.102066 32302935 PMC7151415

[bibr20-0192513X211030028] Reed R. G. O'Connor M. F. Pace T. W. Raison C. L. Butler E. A. (2017). Dyadic coping and salivary interleukin-6 responses to interpersonal stress. Journal of Family Psychology, 31(3), 367. 27668934 10.1037/fam0000249

[bibr21-0192513X211030028] Revenson T. A. Kayser K. E. Bodenmann G. E. (2005). Couples coping with stress: Emerging perspectives on dyadic coping. American Psychological Association.

[bibr22-0192513X211030028] Røsand G. M. B. Slinning K. Eberhard-Gran M. Røysamb E. Tambs K. (2012). The buffering effect of relationship satisfaction on emotional distress in couples. BMC Public Health, 12(1), 1-13. 22264243 10.1186/1471-2458-12-66 PMC3295683

[bibr23-0192513X211030028] Schmaling K. B. Afari N. Barnhart S. Buchwald D. S. (1997). The association of disease severity, functional status, and medical utilization with relationship satisfaction among asthma patients and their partners. Journal of Clinical Psychology in Medical Settings, 4(4), 373-382.

[bibr24-0192513X211030028] Scott S. B. Rhoades G. K. Stanley S. M. Allen E. S. Markman H. J. (2013). Reasons for divorce and recollections of premarital intervention: Implications for improving relationship education. Couple and Family Psychology: Research and Practice, 2(2), 131–145. DOI: 10.1037/a0032025 24818068 PMC4012696

[bibr25-0192513X211030028] Scott M. E. Schelar E. Manlove J. Cui C. (2009, July). Young adult attitudes about relationships and marriage: Times may have changed, but expectations remain high*.* Childtrends: Childtrends.org.

[bibr26-0192513X211030028] Waddell N. Overall N. C. Chang V. T. Hammond M. D. (2021). Gendered division of labor during a nationwide COVID-19 lockdown: Implications for relationship problems and satisfaction. Journal of Social and Personal Relationships, 38(6), 1759-1781.

[bibr27-0192513X211030028] Waite L. J. Gallagher M. (2001). The case for marriage: Why married people are happier, healthier, and better off financially. Random House Digital, Inc.

[bibr28-0192513X211030028] Wienke C. Hill G. J. (2009). Does the “Marriage Benefit” extend to partners in Gay and Lesbian Relationships?: Evidence from a random sample of sexually active adults. Journal of Family Issues, 30(2), 259–289. DOI: 10.1177/0192513x08324382

[bibr29-0192513X211030028] Wildsmith E. Manlove J. Steward-Streng N. Cook E. (2013, December). The dynamics in young adult romantic relationships*.* Childtrends: childtrends.org

[bibr30-0192513X211030028] Xiang Y. Yang Y. Li W. Zhang L. Zhang Q. Cheung T. Ng C. H. (2020). Timely mental health care for the 2019 novel coronavirus outbreak is urgently needed. Lancet, 7, 228–229. DOI: 10.1016/S2215-0366(20)30046-8 PMC7128153 32032543

[bibr31-0192513X211030028] Zhang W. Wang K. Yin L. Zhao W. Xue Q. Peng M. Wang H. (2020). Mental Health and Psychosocial Problems of Medical Health Workers during the COVID-19 Epidemic in China. Psychotherapy and Psychosomatics, 89(4), 242–250. DOI: 10.1159/000507639 32272480 PMC7206349

